# Mechanism of OTUD5 in non-small cell lung cancer cell proliferation, invasion, and migration

**DOI:** 10.17305/bjbms.2022.7206

**Published:** 2022-06-27

**Authors:** Xuebing Li, Baohua Lu, Lina Zhang, Jing Yang, Yurong Cheng, Dong Yan

**Affiliations:** 1 Department of Oncology, Beijing Luhe Hospital Affiliated to Capital Medical University, Beijing, China; 2 Department of Oncology, Beijing Chest Hospital, Capital Medical University, Beijing, China; 3 Department of Cellular and Molecular Biology, Beijing Chest Hospital, Capital Medical University, Beijing Tuberculosis and Thoracic Tumor Research Institute, Beijing, China

**Keywords:** Invasion, miR-652-3p, non-small cell lung cancer, ovarian tumor protease deubiquitinase 5, phosphatase and tensin homolog, proliferation, migration, ubiquitination

## Abstract

Ovarian tumor protease deubiquitinase 5 (OTUD5) has been discussed as a regulator of cancer development. Herein, the present study set out to explore the molecular mechanism of OTUD5 in non-small cell lung cancer (NSCLC) cell proliferation, invasion, and migration. First, the expression patterns of OTUD5, phosphatase, and tensin homolog (PTEN), as well as microRNA (miR)-652-3p in cells were detected by qRT-PCR and Western blot. Cell viability, migration, and invasion were assessed with the help of cell-counting kit-8 and Transwell assays, in addition to the measurement of the ubiquitination and protein levels of PTEN. The binding relations between OTUD5 and PTEN, and miR-652-3p and OTUD5 were testified by coimmunoprecipitation or dual-luciferase assays. Cells were further treated with GSK2643943A (inhibitor of deubiquitinase) or miR-652-3p-inhibitor to explore the role of PTEN ubiquitination and miR-652-3p in NSCLC cells. OTUD5 and PTEN were both poorly-expressed, and miR-652-3p was highly-expressed in NSCLC cells. On the other hand, over-expression of OTUD5 suppressed NSCLC cell proliferation, invasion, and migration. OTUD5 deubiquitinated and stabilized PTEN, and miR-652-3p targeted and inhibited OTUD5 expression. Augmenting the ubiquitination levels of PTEN promoted NSCLC cell growth, whereas miR-652-3p inhibition promoted the tumor-suppressing effects of the OTUD5/PTEN axis in NSCLC. Altogether, our findings highlighted that miR-652-3p restrained the role of OTUD5 in deubiquitinating PTEN to improve PTEN protein level, thereby promoting NSCLC cell proliferation, invasion, and migration.

## INTRODUCTION

Non-small cell lung cancer (NSCLC), the most prevalent form of lung cancer (LC), is primarily classified into two subtypes, namely, lung adenocarcinoma and squamous cell carcinoma [1]. NSCLC treatment is improved with the clinical implementation of targeted therapies, including the adoption of small-molecule tyrosine kinase inhibitors and antibodies targeting T-cell receptor programmed cell death-1 and its ligand [2,3]. Despite the advent of such therapeutic modalities, the prognoses of NSCLC patients remain dismal, which can be attributed to late diagnosis and high metastatic potential of these tumors [4]. Therefore, it is prudent to advance the search for effective molecules that could target NSCLC cell growth and metastasis to reduce the plight of NSCLC.

The process of ubiquitination, a ubiquitin-mediated protein post-translational modification, carries out the critical function of regulating protein degradation, localization, or function, whereas deubiquitinases (DUBs) reverse ubiquitination by removing ubiquitin from ubiquitinated proteins [5,6]. Together, ubiquitination and deubiquitination, induced by ubiquitin ligases and DUBs, are involved in the stabilization of cancer-related proteins and altering their oncogenic or anti-oncogenic functions [7-10]. Meanwhile, the superfamily of ovarian tumor protease deubiquitinases (OTUD) plays a role in a myriad of biological processes, including virus infection, bone remodeling, tumorigenesis, stem cell differentiation, and DNA repairing [11]. What’s noteworthy, OTUD5, a member of the OTUD superfamily, was previously shown to serve as a tumor suppressor in NSCLC by deubiquitinating anti-oncogenes, including Tripartite motif 5, p53, and programmed cell death 5 [12,13].

Another key focus of the present study, phosphatase and tensin homolog (PTEN), has emerged as a well-established tumor suppressor gene, such that its inactivation or mutation is implicated in a plethora of malignancies, such as breast, thyroid, colon, and endometrial cancers [14,15]. Moreover, PTEN is known to be capable of negatively regulating the PI3K/mTOR/Akt oncogenic pathway, thus conferring a profound role in targeted therapy of LC [16]. Interestingly, ubiquitination/deubiquitination serves as one of the major regulatory mechanisms of PTEN in cancer by influencing its stability, subcellular localization, and activity [17]. Furthermore, the study performed by Qin et al. revealed that ubiquitination-mediated degradation of PTEN hijacks cell growth rheostat control for neoplastic remodeling [18]. However, it has not been reported whether OTUD5 can stabilize PTEN by deubiquitination, and thereby influence the fate of NSCLC cells.

MicroRNAs (miRNAs), a class of small transcripts with about 22 nucleotides, are well-known to be dysfunctional in tumorigenesis, in addition to their roles in cancer diagnosis and tailored therapy [19]. What’s more, various miRNAs have been identified as promising targets for lung cancer treatment [20]. In addition, some miRNAs possess the ability to interact with ubiquitin ligases or DUBs to form feedback loops that regulate cancer development [21-24]. Therefore, exploring the interaction between miRNA and ubiquitin-proteasome system may provide a novel strategy for efficacious treatment of NSCLC. Of note, miR-652-3p, a well-documented onco-miRNA, was previously documented to be up-regulated in NSCLC and facilitate NSCLC proliferation and metastasis [25]. Herein, initial database prediction and a dual-luciferase assay revealed that miR-652-3p functions as an upstream target of OTUD5. Nevertheless, whether miR-652-3p regulates that the OTUD5/PTEN axis has not been discussed before and warrants further exploration.

In light of the abovementioned evidence, we put forth a hypothesis that OTUD5 deubiquitinates and stabilizes PTEN to suppress NSCLC cell proliferation, invasion, and migration, whereas this mechanism could be counteracted by miR-652-3p overexpression. Consequently, the present study was carried out to unveil the role of miR-652-3p/OTUD5/PTEN and their interactions in NSCLC and provide a novel theoretical basis for NSCLC treatment.

## MATERIALS AND METHODS

### Cell culture

Human bronchial epithelioid cell lines 16HBE (procured from Millipore, Bedford, MA, USA) and NSCLC cell lines A549 and NCI-H292 (procured from ATCC, Manassas, VA, USA) and PC9 cells (procured from Tongpai Biotechnology Co., Ltd, Shanghai, China) were cultured in Roswell Park Memorial Institute 1640 medium (RPMI) containing 10% fetal bovine serum (FBS), 100 μg/ml streptomycin, and 100 U/ml penicillin (Invitrogen, Carlsbad, CA, USA) in a 5% CO2 incubator at 37°C.

### Cell transfection

First, pcDNA3.1-OTUD5, miR-652-3p-inhibitor, and their control plasmids were all supplied by GenePharma (Shanghai, China). In accordance with the provided instructions, the Lipofectamine 3000 reagent (Invitrogen, Carlsbad, CA, USA) was adopted to transfect the above plasmids into A549 or NCI-H460 cells. Meanwhile, 160 nM GSK2643943A (MedChemExpress Co., Ltd., Monmouth Junction, NJ, USA) or 100 nM MG132 (MCE) was added to the cell culture medium, with equal amounts of dimethylsulfoxide serving as the control.

### Cell counting kit-8 (CCK-8) assay

CCK-8 assay was carried out to assess cell viability in accordance with the manufacturer’s instructions. Briefly, transfected cells (N = 2.0 × 10^3^) were seeded into 96-well plates. At 24 h, 48 h, and 72 h post cell seeding, the cells were treated with 10 μL CCK-8 solution. Afterward, the cells were cultured at 37°C for 2.5 h, followed by absorbance measurement at a wavelength of 450 nm using a microplate reader.

### Transwell assays

Transwell assays were carried out in accordance with a previously published method [26]. In brief, about 1 × 10^4^ transfected cells were suspended in 200 mL serum-free culture medium and placed in the apical chamber. The filter was covered with Matrigel (BD Biosciences, San Jose, CA, USA) in the invasion assay, not in the migration assay. The basolateral chamber was supplemented with RPMI 1640 medium containing 10% FBS as the chemical attractant. Cells were subsequently subjected to 48-h culture for the invasion assay and 24-h culture for the migration assay. Next, cells in the apical chamber were removed using cotton swabs, and cells on the lower surface of the filter were fixed with 0.1% crystal violet. Afterward, the number of cells on the optical filter in three random areas was counted with the help of an optical microscope (Olympus, Tokyo, Japan).

### Quantitative real-time reverse transcriptase polymerase chain reaction (qRT-PCR)

In accordance with the manufacturer’s instructions, total RNA content was extracted from cells using the TRIzol reagent. Complementary DNA was synthesized by PrimeScript reverse transcription kits (Invitrogen), and qRT-PCR was conducted with SYBR Premix Ex Taq II (Takara, Tokyo, Japan) and a 7500 real-time RT PCR system (Applied Biosystems, Inc., Carlsbad, CA, USA). Primer sequences are illustrated in Table 1. GAPDH and U6 were adopted as standardized controls of OTUD5 mRNA and miR-652-3p, respectively [27]. The relative gene expression was calculated by means of the 2-ΔΔCt method.

**TABLE 1:**
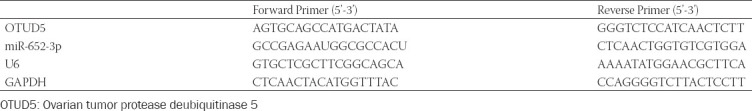
qPCR primers

### Western blot assay

Total protein content was extracted from cells using a radioimmunoprecipitation assay lysis buffer (Invitrogen) and quantified with the bicinchoninic acid method. Subsequently, 50 μg proteins were subjected to sodium dodecyl sulfate-polyacrylamide gel electrophoresis (SDS-PAGE), and then transferred to nitrocellulose membranes. The membranes were subsequently blockaded with 5% non-fat milk and incubated with antibodies anti-OTUD5 (dilution ratio of 1:2000, ab176727, Abcam, Cambridge, MA, USA), anti-PTEN (dilution ratio of 1:1000, ab267787, Abcam), and anti-GAPDH (dilution ratio of 1:1000, ab9485, Abcam) at 4°C overnight. Following rinsing with Tris Buffered Saline Tween (TBST) thrice (15 min each time), the membranes were cultured with horseradish peroxidase (HRP)-coupled secondary antibody (dilution ratio of 1:2000, ab6721, Abcam) at room temperature for 1 h. Afterward, the membranes were washed with TBST thrice (15 min each time), reacted with an enhanced electrochemical luminescence reagent, and imaged. The gray value was analyzed using the Image J software, with GAPDH serving as the internal reference.

### Coimmunoprecipitation (Co-IP)

Cells were re-suspended in the lysis buffer (50 nM Tris-HCl pH = 7.6–8.0, 0.5% NP40, 1 nM ethylene diamine tetraacetic acid, and 1 mM b-mercaptoethanol) containing a protease inhibitor, and then subjected to ultrasonic lysing. Following 10-min centrifugation at 14000 g, the supernatant was incubated overnight with antibody anti-PTEN (ab267787, Abcam) or anti-IgG (ab172730, Abcam) and protein A/G agarose beads at 4°C overnight. After washing with the lysis buffer thrice, the beads were boiled in 40 μL 2 × SDS-PAGE sample buffer and then subjected to SDS-PAGE, followed by Western blot assay with the anti-OTUD5 (ab176727, Abcam) and HRP-coupled secondary antibody (ab6721, Abcam). Afterward, the protein bands were visualized using the chemiluminescence method.

### Ubiquitination assay

Ubiquitination assay was carried out following a previously published method [28]. Briefly, the cells were resuspended in the lysis buffer containing protease inhibitor and subjected to ultrasonic lysing. Following 10-min centrifugation at 15000 g, the supernatant was incubated with anti-PTEN (ab267787, Abcam) or anti-IgG (ab172730, Abcam) antibody-coupled agarose beads at 4°C and rotated overnight. After extensive washing, the beads were boiled in 2 × loading buffer for 5 min and isolated using SDS-PAGE, followed by Western blot assay with the anti-Ub antibody (ab6721, Abcam).

### Bioinformatics analysis

The expression pattern of OTUD5 in NSCLC was predicted with the help of the GEPIA database (http://gepia.cancer-pku.cn/) [29]. The downstream miRNAs of OTUD5 were predicted with the help of the StarBase database (https://starbase.sysu.edu.cn/) [30]. In addition, the binding sites of miR-652-3p and OTUD5 were also predicted using the StarBase database.

### Dual-luciferase assay

Wild or mutant types of OTUD5 3’ UTR sequences containing complementary sites of miR-652-3p were inserted into pmiGLO vectors (Promega, Madison, WI, USA) to construct the wild-type luciferase reporter plasmid (OTUD5 3’UTR-WT) and the mutant derivative (OTUD5 3’UTR-MUT). In accordance with the provided instructions, the aforementioned luciferase reporter plasmids were cotransfected into A549 or HCI-H460 cells with miR-652-3p-mimic or mimic-NC using the Lipofectamine 3000 reagent (Invitrogen). Afterward, the relative luciferase activity was analyzed with the help of the dual-luciferase assay system (Promega).

### Statistical analysis

Data analyses and graphing were performed using the GraphPad Prism 8.0 software (GraphPad Software Inc., San Diego, CA, USA). Measurement data were represented as mean ± standard deviation (SD). Analysis of pairwise comparisons were processed using the t test and those of multi-group comparisons were processed by means of one-way or two-way analysis of variance (ANOVA), followed by Tukey’s *post-hoc* test. A value of *p* < 0.05 was indicative of statistical significance.

## RESULTS

### OTUD5 is poorly-expressed in NSCLC

**FIGURE 1:**
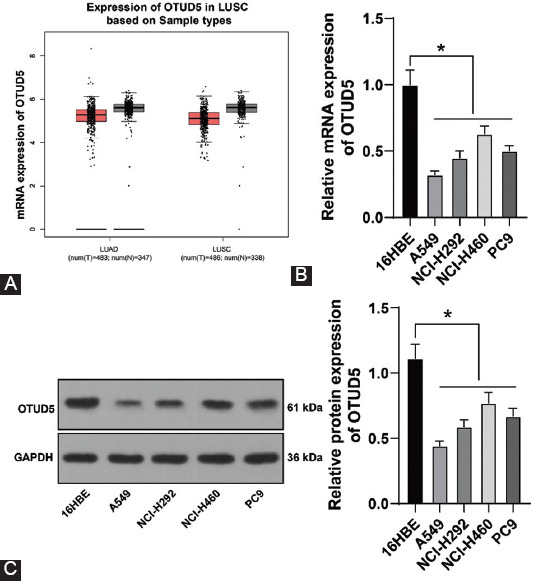
Ovarian tumor protease deubiquitinase 5 (OTUD5) is poorly-expressed in NSCLC. (A) OTUD5 expression in NSCLC predicted by the GEPIA database; (B and C) mRNA and protein levels of OTUD5 detected by qRT-PCR and Western blot. Experiments were performed three times independently. Data were represented as mean ± SD. Data in figures B and C were analyzed using one-way ANVOA, followed by Tukey’s *post hoc* test. *p < 0.05. T: Tumor tissue; N: Normal tissues.

To analyze the role of OTUD5 in NSCLC, we first determined the expression patterns of OTUD5 in NSCLC. The GEPIA database predicted that OTUD5 was poorly expressed in NSCLC (Figure 1A). Subsequently, we detected OTUD5 expression levels in human bronchial epithelioid cells 16HBE and NSCLCs (A549, NCI-H292, NCI-H460, and PC9) using qRT-PCR and Western blot analysis, the results of which revealed that OTUD5 expression levels in NSCLC cells were significantly lower compared to those in 16HBE cells (*p* < 0.05, Figure 1B and C). Together, these findings highlighted that OTUD5 was poorly-expressed in NSCLC.

### Overexpression of OTUD5 suppresses NSCLC cell proliferation, invasion, and migration

**FIGURE 2:**
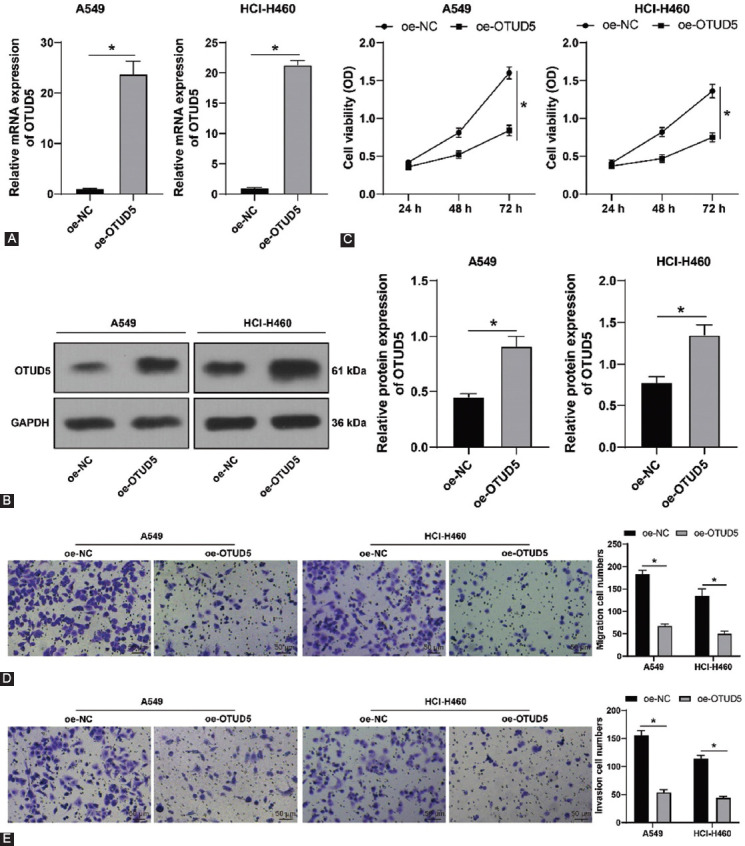
Ovarian tumor protease deubiquitinase 5 (OTUD5) overexpression suppresses NSCLC cell proliferation, invasion, and migration. A549 and HCI-H460 cells were transfected with pcDNA3.1-OTUD5 (oe-OTUD5), with pcDNA3.1-NC (oe-NC) as the negative control. (A and B) OTUD5 expressions in A549 and HCI-H460 cells detected by qRT-PCR and Western blot; (C) cell viability of A549 and HCI-H460 cells assessed by CCK-8 assay; (D and E) number of migrated and invaded A549 and HCI-H460 cells determined by Transwell assays. Experiments were performed 3 times independently. Data were represented as mean ± SD. Data in Figures A and B were analyzed using the *t*-test and data in figures C-E were analyzed using two-way ANOVA, followed by Tukey’s *post hoc* test. *p < 0.05.

To further explore the influence of OTUD5 on NSCLC cell proliferation, migration, and invasion, we selected A549 cells presenting with the lowest OTUD5 expression, and HCI-H460 cells presenting with the highest OTUD5 expression for further analyses. A549 and HCI-H460 cells were transfected with pcDNA3.1-OTUD5 to over-express OTUD5 (*p* < 0.05, Figure 2A and B). Subsequent results elicited that cell viability of A549 and HCI-H460 cells was significantly decreased following OTUD5 overexpression (*p* < 0.05, Figure 2C), in addition to a decline in the number of migrated and invaded cells (*p* < 0.05, Figure 2D and E). Overall, these findings suggested that overexpression of OTUD5 suppressed NSCLC cell proliferation, invasion, and migration.

### OTUD5 stabilizes PTEN through deubiquitination

**FIGURE 3:**
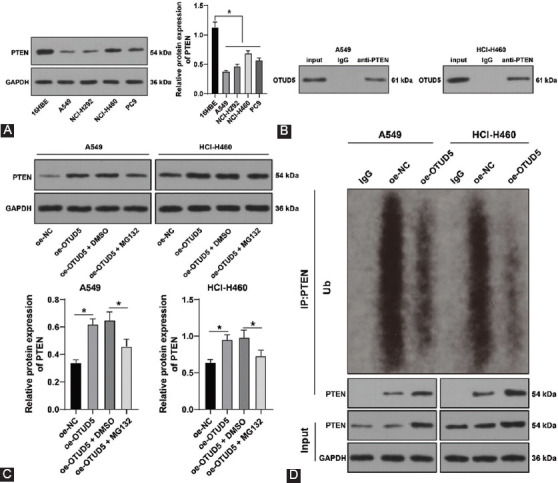
Ovarian tumor protease deubiquitinase 5 (OTUD5) stabilizes phosphatase and tensin homolog (PTEN) through deubiquitination. (A) Protein levels of PTEN in NSCLC cell lines detected by Western blot; (B) the binding relationship between OTUD5 and PTEN detected by Co-IP assay; (C) protein levels of PTEN in A549 and HCI-H460 cells detected by Western blot; (D) ubiquitination levels of PTEN in A549 and HCI-H460 cells. Experiments were performed three times independently. Data were represented as mean ± SD. Data in figures A and C were analyzed using one-way ANOVA, followed by Tukey’s *post hoc* test. *p < 0.05.

Thereafter, we explored the downstream target genes of OTUD5 and their respective influence in regard to NSCLC. OTUD5, as a deubiquitinating enzyme, is capable of stabilizing the protein level by means of deubiquitination [31]. Existing evidence further suggests that PTEN can be deubiquitinated to stabilize its expression [32], while PTEN is also known to be poorly-expressed in NSCLC [33]. Accordingly, we detected the protein levels of PTEN in NSCLC cells with a Western blot assay, which revealed that PTEN protein levels were significantly down-regulated in NSCLC cells (*p* < 0.05, Figure 3A). Therefore, we conjectured that OTUD5 stabilizes the protein level of PTEN through deubiquitination. Subsequent results of Co-IP assay demonstrated the presence of a binding relationship between OTUD5 and PTEN (Figure 3B). We further detected the protein levels of PTEN in A549 and HCI-H460 cells with another Western blot assay and uncovered that overexpression of OTUD5 promoted the protein level of PTEN, while PTEN protein levels were further increased on treatment with MG132 (a proteasome inhibitor) (*p* < 0.05, Figure 3C), indicating that OTUD5 may regulate PTEN expression through the proteasome pathway. Furthermore, we quantified the ubiquitination levels of PTEN in A549 and HCI-H460 cells and documented that OTUD5 overexpression reduced the ubiquitination level of PTEN (Figure 3D). Altogether, these findings indicated that OTUD5 stabilized the protein levels of PTEN through deubiquitination.

### Increasing ubiquitination level of PTEN reverses the inhibition of OTUD5 overexpression on NSCLC cell proliferation, invasion, and migration

**FIGURE 4:**
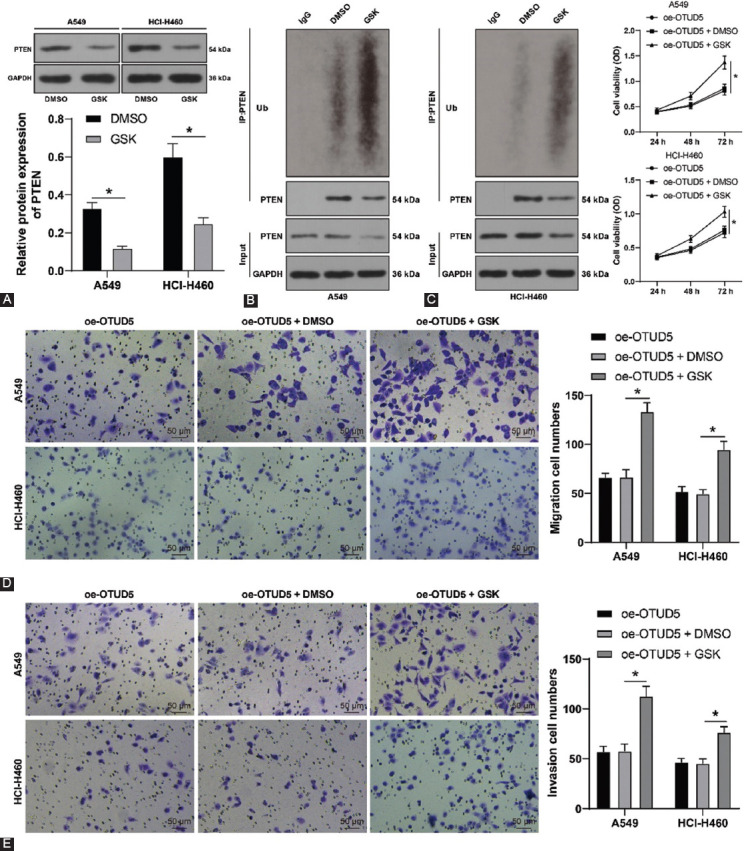
Increasing the ubiquitination level of phosphatase and tensin homolog (PTEN) reverses the inhibition of Ovarian tumor protease deubiquitinase 5 (OTUD5) overexpression on NSCLC cell proliferation, invasion, and migration. A549 and HCI-H460 cells were treated with GSK2643943A (GSK), with DMSO treatment as the control. (A) Protein levels of PTEN detected by Western blot; (B) ubiquitination levels of PTEN, followed by a collaborative experiment with oe-OTUD5; (C) cell viability assessed by CCK-8 assay; (D and E) number of migrated and invaded cells determined by Transwell assay. Experiments were performed three times independently. Data were represented as mean ± SD. Data in figures A and C, and E were analyzed using two-way ANOVA, followed by Tukey’s *post hoc* test. *p < 0.05.

To further validate that OTUD5 executes deubiquitination to regulate NSCLC cell proliferation, invasion, and migration, the ubiquitination levels in A549 and HCI-H460 cells were enhanced using GSK2643943A (GSK). Subsequent experimentation elicited that the protein levels of PTEN were significantly declined (*p* < 0.05, Figure 4A), while the ubiquitination levels of PTEN were significantly increased (*p* < 0.05, Figure 4B) as a result of GSK treatment. Thereafter, we carried out a collaborative experiment with a combination of GSK and pcDNA3.1-OTUD5 treatments and uncovered that cell viability of A549 and HCI-H460 cells was significantly enhanced (*p* < 0.05, Figure 4C), and the number of migrated and invaded cells was augmented (*p* < 0.05, Figure 4D and E) following GSK treatment. Overall, these findings suggested that augmenting the ubiquitination level of PTEN reversed the inhibition of OTUD5 overexpression on NSCLC cell proliferation, invasion, and migration.

### miR-652-3p inhibits OTUD5 expression

**FIGURE 5:**
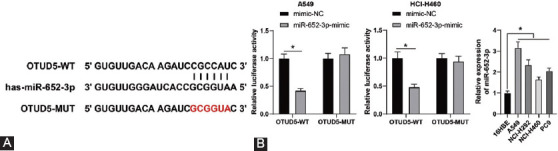
miR-652-3p inhibits OTUD5 expression. (A) Binding relationship between miR-652-3p and OTUD5 testified by the dual-luciferase assay; (B) miR-652-3p expression in NSCLC cell lines detected by qRT-PCR. Experiments were performed three times independently. Data were represented as mean ± SD. Data in Figure A were analyzed using two-way ANOVA, and data in figure B were analyzed using one-way ANOVA, followed by Tukey’s *post hoc* test. *p < 0.05.

Furthermore, we explored the upstream target genes of OTUD5. The study carried out by Bai et al. reported that OTUD5 expression can be inhibited by its target miRNAs [34]. We predicted the upstream miRNAs of OTUD5 on the StarBase database and focused our efforts on miR-652-3p. Interestingly, miR-652-3p was previously documented to be highly-expressed in NSCLC [25]. Accordingly, we postulated that miR-652-3p serves as an upstream target of OTUD5. Subsequent dual-luciferase assay revealed a binding relationship between miR-652-3p and OTUD5 3’UTR (*p* < 0.05, Figure 5A). In addition, we detected miR-652-3p expression patterns in NCSLC cell lines by means of qRT-PCR, which revealed that miR-652-3p was strongly expressed in NSCLC cells (*p* < 0.05, Figure 5B). Altogether, these findings indicated that miR-652-3p served as an upstream target of OTUD5 and miR-652-3p was highly-expressed in NSCLC cells.

### Downregulation of miR-652-3p suppresses NSCLC cell proliferation, invasion, and migration through the OTUD5/PTEN axis

**FIGURE 6:**
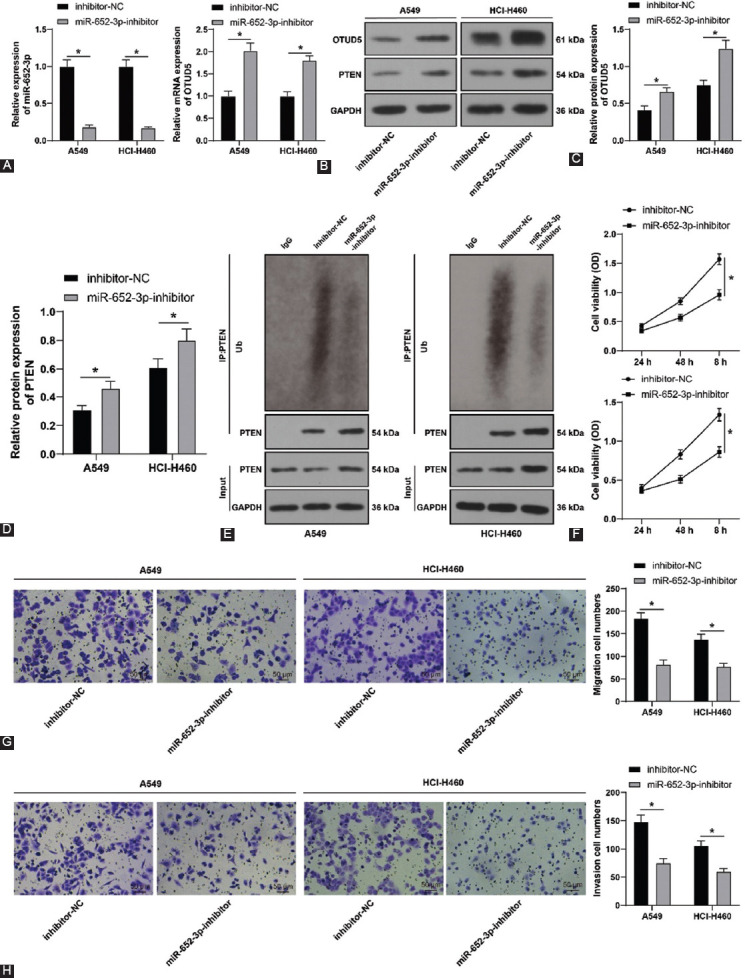
miR-652-3p downregulation suppresses NSCLC cell proliferation, invasion, and migration through the ovarian tumor protease deubiquitinase 5 (OTUD5)/phosphatase and tensin homolog (PTEN) axis. A549 and HCI-H460 cells were transfected with miR-652-3p-inhibitor, with inhibitor-NC as the negative control. (A) miR-652-3p expression detected by qRT-PCR; (B and C) OTUD5 expression detected by qRT-PCR and Western blot; (D) protein level of PTEN detected by Western blot; (E) ubiquitination level of PTEN; (F) cell viability assessed by CCK-8 assay; (G and H) number of migrated and invaded cells determined by Transwell assay. Data in figures A-H were analyzed using two-way ANOVA, followed by Tukey’s *post hoc* test. *p < 0.05.

Finally, we explored whether miR-652-3p regulated NSCLC cell proliferation, invasion, and migration through the OTUD5/PTEN axis. Briefly, A549 and HCI-H460 cells were transfected with miR-652-3p-inhibitor to inhibit miR-652-3p expression (*p* < 0.05, Figure 6A). Subsequent experimentation revealed that miR-652-3p inhibition brought about an increase in OTUD5 expression levels (*p* < 0.05, Figure 6B and C) and PTEN protein levels (*p* < 0.05, Figure 6D and E), while simultaneously reducing the ubiquitination level of PTEN, cell viability (*p* < 0.05, Figure 6F), and the number of migrated and invaded cells (*p* < 0.05, Figure 6G and H). Collectively, these findings suggested that downregulation of miR-652-3p suppressed NSCLC cell proliferation, invasion, and migration through the OTUD5/PTEN axis.

## DISCUSSION

NSCLC, the major subtype of LC, contributes to high mortality and morbidity rates across the globe [25]. Meanwhile, the hard-done work of our peers has shown that deubiquitinases (DUBs) play a key role in cancer progression by improving protein stability [35-37]. Moreover, some miRNAs possess the ability to function as regulators of DUBs to alter the ubiquitination levels of cancer-related proteins and further influence the malignant characteristics of cancer cells [24,38]. Herein, we carried out a series of experiments to uncover the role of miR-652-3p/OTUD5/PTEN and their interactions in NSCLC, in an effort to provide a novel theoretical basis for NSCLC treatment. The obtained findings revealed that OTUD5 deubiquitinated and stabilized PTEN to suppress NSCLC proliferation, invasion, and migration, and miR-652-3p serving as an upstream target of OTUD5 abolished the tumor-suppressing role of OTUD5/PTEN in NSCLC.

There is a plethora of evidence to highlight the correlation between decreased levels of OTUD5 and poor prognoses in NSCLC patients, such that OTUD5 knockdown exerts an enhancing effect on NSCLC cell proliferation, migration, and chemoresistance [13]. On the other hand, high expression of OTUD5 was associated with improved overall survival in Stage II NSCLC, further underscoring the beneficial role of OTUD5 for tumor repression in NSCLC [39]. In our study, we came across downregulated levels of OTUD5 in NSCLC cell lines (A549, NCI-H292, NCI-H460, and PC9), which is in line with the prediction results from the GEPIA database. To elaborate our understanding of OTUD5 functionality in NSCLC cells, we further overexpressed OTUD5 in A549 and HCI-H460 cells and learnt that OTUD5 overexpression led to a reduction in cell viability, migration, and invasion. In accordance, OTUD5 is also negatively correlated with clinicopathologic characteristics of liver and cervical cancers [12,34]. In lieu of these findings and evidence, it would be plausible to suggest that OTUD5 suppresses NSCLC cell proliferation, invasion, and migration.

The process of ubiquitination is known to serve as a vital post-translational modification and consequently exerts control over tumor-suppressing and tumor-promoting proteins in cancer [40]. OTUD5 as a DUB removes ubiquitin from target proteins, thus reversing ubiquitin-dependent degradation and improving protein stability [41,42]. More importantly, DUBs, such as ubiquitin-specific protease 10, 13, and 20, have been previously documented to restore the protein stability of PTEN in cancers [43-46]. The latter is particularly important as PTEN is lauded as a promising therapeutic target in cancer therapy [47-49]. Additional experimentation in our study illustrated the down-regulation of PTEN protein levels in NCSLC cells and validated the binding relationship between OTUD5 and PTEN. Besides, a prior study demonstrated that OTUD5 overexpression and MG132 treatment bring about an elevation in the protein levels of PTEN in A549 and HCI-H460 cells, along with decreased ubiquitination levels of PTEN, suggesting that OTUD5 stabilized PTEN by means of deubiquitination.

Thereafter, we promoted the total ubiquitination levels in A549 cells with the help of GSK2643943A (GSK), which led to decreased PTEN protein levels and increased ubiquitination levels. In addition, a collaborative experiment was carried out with GSK and pcDNA3.1-OTUD5 in A549 cells and brought about an enhancement in cell viability, migration, and invasion. In accordance with our findings, there is much evidence to suggest that epigenetic silencing of PTEN facilitates NCSLC cell growth, mobility, and chemoresistance [50-52]. Besides, elevated ubiquitination or degradation of PTEN was previously shown to drive the progression of bladder, prostate, brain, and pancreatic cancers [53-56]. Altogether, the abovementioned findings and valuable evidence highlighted that OTUD5 exerts an anti-tumor function by means of deubiquitinating and stabilizing PTEN, while enhancing the ubiquitination of PTEN reverses the inhibition of OTUD5 overexpression on NSCLC progression.

Furthermore, we focused our efforts on the upstream targets of OTUD5. A prior bioinformatics analysis reported that a number of miRNAs, such as miR-137, miR-1913, miR-937, miR-607, and miR-3149, are capable of negatively-regulating the mRNA expression of OTUD5 [34]. In our study, one such mRNA, namely, miR-652-3p, was predicted as an upstream miRNA of OTUD5, which was further validated by means of a dual-luciferase assay. Moreover, there is also evidence to suggest that miR-652-3p is involved in the oncogenic ceRNA network for NSCLC, which further emphasizes its participation in the pathogenesis of NSCLC [57]. Hence, we silenced miR-652-3p expression in A549 cells with a miR-652-3p-inhibitor and uncovered that silencing miR-652-3p led to a reduction in NSCLC cell viability, migration, and invasion, along with increased OTUD5 and PTEN protein levels and diminished ubiquitination levels.

Consistently, a prior study reported that miR-652-3p is highly-expressed in NSCLC, such that overexpressed miR-652-3p accelerates cell proliferation and metastasis and restricts apoptosis by modulating Lgl1 [25]. Above all, our findings demonstrated that miR-652-3p may abolish the tumor-suppressing role of OTUD5/PTEN, thereby facilitating NSCLC cell proliferation, invasion, and migration.

## CONCLUSION

In summary, our study was the first-of-its-kind to shed light on the interplay of miR-652-3p/OTUD5/PTEN in NSCLC cells and provide novel insight into the possible application of OTUD5 in NSCLC treatment. However, we failed to perform analysis of clinical samples or *in vivo* assays with xenograft tumors. In addition, except OTUD5 and miR-652-3p, it remains unknown whether other DUBs and upstream miRNAs of OTUD5 are involved with NSCLC cell behaviors, which requires further experimentation. We shall strive to validate our conclusions with clinical analysis, *in vivo* assays, and experiments about other DUBs in our future endeavors.
